# The efficacy of exercise in preventing injury in adult male football: a systematic review of randomised controlled trials

**DOI:** 10.1186/s40798-014-0004-6

**Published:** 2015-01-20

**Authors:** Tom Porter, Alison Rushton

**Affiliations:** School of Sport, Exercise and Rehabilitation Sciences, College of Life and Environmental Sciences, University of Birmingham, Edgbaston, Birmingham B15 2TT UK

## Abstract

**Background:**

Injury prevention measures might reduce the impact of injury on footballers and football clubs. Increasing research has evaluated the use of exercise for injury prevention. However, research has focused on adolescent females. No high-quality systematic reviews have evaluated the efficacy of all forms of exercise on preventing injury in adult male football.

**Objective:**

Our objective was to conduct a systematic review to evaluate the efficacy of exercise in preventing injury in adult male football.

**Data sources:**

Comprehensive searches of electronic databases CINAHL (Cumulative Index to Nursing and Allied Health Literature), MEDLINE, Embase, AMED (The Allied and Complementary Medicine Database), the Cochrane Central Register of Controlled Trials, PEDro (The Physiotherapy Evidence Database), SPORTDiscus™, the National Research Register, Current Controlled Trials website (York), and http://www.ClinicalTrials.gov were conducted using predefined search terms to identify relevant studies published up to 1 March 2013. Screening of references, searches of grey literature, and hand searches of relevant journals were also employed.

**Study selection:**

Included studies were randomized controlled trials using injury incidence as an outcome measure to evaluate the efficacy of an exercise intervention on uninjured male footballers aged 16 years and over. Articles not written in English were excluded.

**Data extraction:**

Two researchers independently searched data sources, screened studies for eligibility, evaluated risk of bias, and extracted data using predefined criteria.

**Study appraisal and synthesis methods:**

Risk of bias of included trials was assessed using the Cochrane Collaboration’s tool for assessing risk of bias. There was insufficient trial comparability (outcome measures, interventions, injury type) for meta-analysis, and a qualitative analysis was performed.

**Results:**

Eight trials (*n* = 3,355) from five countries met the inclusion criteria. All trials were assessed as having a high risk of bias. Two trials reported statistically significant reductions in hamstring injuries with eccentric exercise, and two reported statistically significant reductions in recurrent ankle sprains with proprioceptive exercise. Four trials showed no statistically significant difference in injury incidence with exercise interventions targeting a range of injuries.

**Limitations:**

Notable limitations of included trials included poor reporting and limited blinding. A high risk of bias and insufficient comparability across trials prevented quantitative data synthesis.

**Conclusions:**

Limitations in the context of study quality and heterogeneity resulted in an inability to reach a clear conclusion regarding efficacy of exercise for injury prevention in adult male football. Future low risk of bias, properly powered, and comprehensively reported trials are warranted to evaluate the efficacy of exercise on injury prevention. The use of eccentric hamstring exercise for hamstring injury prevention and proprioceptive training for recurrent ankle sprain prevention might be a good focus for future trials, as existing trials with a high risk of bias suggest an effect.

## Key points

- The use of eccentric hamstring exercise may be efficacious in hamstring injury prevention.
- Proprioceptive training may be efficacious in recurrent ankle sprain prevention.
- A lack of high-quality randomised controlled trials of injury prevention in the adult male football population is contradictory to the number of publications on injury epidemiology and risk factors in this population.

## Introduction

### Rationale

Football (soccer) has been described as the world’s most popular sport, with over 265 million registered players [1]. Injury is a major factor influencing player availability up to the highest level of the sport [2,3]. Top European clubs have a mean of 14% of a squad unavailable due to injury at any one time, with players experiencing a mean of two injuries a season [4,5]. This could have a significant impact on team performance and results [6], which in turn could have financial implications for clubs [7,8].

The impact of injury on footballers can be multifaceted. Injuries can result in a detraining effect, with loss of physiological adaptations and fitness, and may become psychological burdens for players [9,10]. Some injuries also have long-term implications, such as the risk of developing early-onset osteoarthritis [11]. This implies that injury prevention measures may benefit player wellbeing as well as enhancing a team’s chance of success. Some clubs include exercises aimed at preventing injuries in their training [12].

There has recently been an increase in research evaluating the use of exercise to reduce the risk of injury to athletes. A number of studies demonstrate that neuromuscular training programs may reduce the risk of injury to females, particularly during adolescence [13-16]. A systematic literature review evaluating the prevention of non-contact anterior cruciate ligament (ACL) injuries with neuromuscular training in female athletes reported a prophylactic effect on injury incidence in all of the five included trials [17]. Interestingly, a meta-analysis evaluating the influence of exercise intervention on the risk of ACL injury in a range of sports included trials with participants of both genders and concluded that injury prevention exercises that include a proprioceptive neuromuscular training component may be more effective in males, reporting a risk reduction of 52% in females and 85% in males [18]. This meta-analysis included eight trials; only one trial (a non-randomized controlled trial evaluating the efficacy of proprioceptive exercise on ACL injury incidence in male footballers) included male participants [19]. The absence of randomization in this trial introduced a risk of selection bias [20], and the predominantly female cohorts evaluated in the review limit extrapolation of the findings to males.

Two systematic reviews evaluate the use of exercise for injury prevention specific to football. Both review the efficacy of interventions on players of all ages and either gender. Kirkendall and Dvorak [21] included trials evaluating any exercise-based intervention and concluded that both specific and generic exercise interventions are effective in preventing injuries. The review used these findings to inform the development of an exercise warm-up program. However, the validity of the findings is limited, as the review had several methodological limitations [22]. Notably, the literature search was inadequately reported, narrow search terms were used, only one database was searched, there were no clear eligibility criteria for study selection, and the risk of bias of included trials was not assessed [22,23]. These limitations limit confidence in the review findings.

The more recent review by van Beijsterveldt et al. [24] excluded trials using injury- or joint-specific exercises and reviewed the efficacy of generic exercise interventions. Contradicting evidence of efficacy of these interventions was attributed to differences in study samples, design, and compliance. Included trials focused predominantly on adolescent female footballers. Four of the six included trials investigated only female participants, and one trial included only male participants. Five of the trials assessed footballers under the age of 19 years.

The high proportion of injury-prevention research in adolescent females is likely to be due to their increased risk of severe injury, such as ACL rupture [25-27]. Also, the positive influence of neuromuscular training has been attributed to improvements in kinematics of movement, which may be more apparent in females due to gender-related differences in limb biomechanics, neuromuscular/strength imbalances, and hormonal influences [28-31]. These gender-related differences imply that injury-prevention research in female footballers may not be extrapolated to males. Males represent the largest group of participants in football, accounting for 90% of players internationally, and it has been reported that they may have an injury incidence similar to or higher than that of females [1,32,33]. There are currently no high-quality systematic reviews evaluating the efficacy of all forms of exercise intervention in reducing the risk of injury in adult male footballers.

### Objectives

The objective of this systematic review is to evaluate the efficacy of exercise in preventing injury in adult male football.

### Methods

This systematic review conforms to the Preferred Reporting Items for Systematic Reviews and Meta-Analyses (PRISMA) statement [22].

### Protocol

This systematic review was completed according to pre-defined protocols that followed the method guidelines from the *Cochrane Handbook* [20] and the Centre for Reviews and Dissemination’s *Guidance for Undertaking Reviews in Healthcare* [34].

### Eligibility criteria

The following criteria were used to inform study selection, with reference to participants, interventions, comparators, outcomes, and study design (PICOS) [23,34].

#### Studies

Studies included were randomized controlled trials (RCTs) with at least one comparison group continuing with regular training. Limiting the comparison to regular training rather than non-exercise interventions allowed a pragmatic analysis of the efficacy of exercise used as an alteration or addition to regular training. Articles not written in English were excluded, rather than not included, to ensure that the risk of language bias could be assessed [35,36]. There were no restrictions on publication date up to 1 March 2013.

#### Participants

Male footballers competing at any level of adult football were included [37]. The age used for inclusion was 16 years, as adult teams often include players of this age [8,38-41]. This is also in accordance with the *FIFA Laws of the Game*, which are rules for players aged 16 and over and may be adjusted for those under 16 [37]. To ensure that the efficacy of exercise in injury prevention rather than rehabilitation was evaluated, only footballers uninjured at the start of a study were included. Mixed populations were included if there was a separate analysis of footballers meeting these criteria.

#### Interventions

Interventions were required to be exclusively exercise based. Studies assessing multiple interventions were included if there was a separate analysis of an exclusively exercise-based intervention.

#### Outcome measures

Included were outcome measures determining injury incidence. This could be a measure of injury rate or risk. Three often utilized measures of injury incidence are the incidence of injury per unit of athlete time (incidence rate), the number of injured athletes divided by the number of athletes at risk (epidemiologic incidence proportion), or the number of injuries divided by the number of athletes at risk (clinical incidence) [42].

### Information sources

Electronic databases including CINAHL (Cumulative Index to Nursing and Allied Health Literature), MEDLINE, Embase, AMED (The Allied and Complementary Medicine Database), the Cochrane Central Register of Controlled Trials, PEDro (The Physiotherapy Evidence Database), SPORTDiscus™, the National Research Register, Current Controlled Trials website (York), and http://www.ClinicalTrials.gov were searched for relevant studies. The National Technical Information Service, System for Information on Grey Literature, and Index to Scientific and Technical Proceedings were searched for unpublished research [34]. All searches were conducted using sensitive search strategies to 1 March 2013.

### Literature search

The search strategies were created by one reviewer and modified as required for each database. Table 1 demonstrates an example of search terms used. Screening of references listed in relevant systematic reviews and hand searches of journals that were the source of at least 10% of the relevant articles were also employed. The searches were performed independently by two reviewers.

**Table 1:** **Example of a search strategy (the Medline OvidSP search)**

1	(Football* OR Soccer).ti,ab
2	exp FOOTBALL/
3	exp SOCCER/
4	1 OR 2 OR 3
5	(injur* OR ruptur* OR sprain* OR strain* OR disloc* OR accident* OR trauma* OR tendin* OR tendon* OR tear* OR fractur* OR break*).ti,ab
6	exp WOUNDS AND INJURIES/
7	exp RUPTURE/
8	exp SPRAINS AND STRAINS/
9	exp ACCIDENTS/
10	exp TENDINOPATHY/OR exp TENDON INJURIES/
11	exp ATHLETIC INJURIES/
12	exp SOFT TISSUE INJURIES/
13	exp KNEE INJURIES/
14	exp FRACTURES, BONE/
15	exp ANKLE INJURIES/
16	exp HIP INJURIES/
17	exp BACK INJURIES/
18	5 OR 6 OR 7 OR 8 OR 9 OR 10 OR 11 OR 12 OR 13 OR 14 OR 15 OR 16 OR 17
19	(exercis* OR neuromuscular OR proprio* OR strength* OR stretch* OR “warm-up” OR balance OR flexibility OR training OR intervention* OR kinesiotherapy OR program*).ti,ab
20	exp EXERCISE/OR exp EXERCISE MOVEMENT TECHNIQUES/OR exp EXERCISE THERAPY/OR exp PLYOMETRIC EXERCISE/
21	exp MUSCLE STRETCHING EXERCISES/OR exp PHYSICAL EDUCATION AND TRAINING/
22	19 OR 20 OR 21
23	(prevent* OR protect* OR risk* OR reduc* OR avoid* OR prehab* OR reccuren*).ti,ab
24	exp ACCIDENT PREVENTION/
25	exp RECURRENCE/
26	23 OR 24 OR 25
27	22 OR 26
28	(trial* OR random* OR control* OR rct).ti,ab
29	(trial* OR random* OR control* OR rct).pt
30	exp RANDOMIZED CONTROLLED TRIAL/
31	28 OR 29 OR 30
32	4 AND 18 AND 27
33	31 AND 32

### Study selection

The two reviewers independently screened the title and abstract of all identified studies, assessing the studies for inclusion using the grades ‘eligible’, ‘may be eligible’, or ‘not eligible’ [43] for each criterion on a standardized form. Studies that did not meet the inclusion criteria were excluded. If a study could not be unequivocally excluded, if there was disagreement between reviewers, or if there was insufficient information in the title and abstract, the full text of the study was obtained. The full texts were independently reviewed, and a study was included if both reviewers were satisfied that it met the eligibility criteria. If there was disagreement between the reviewers, agreement was sought by consensus after consulting the protocol, with a third reviewer mediating in the event of on-going disagreement. The level of agreement between reviewers was evaluated using Cohen’s weighted *κ* [44], and using the Landis and Koch criteria for interpretation [45].

### Data collection process

Data from each included trial were independently extracted by the two reviewers using a standardized form based on the guidance in the *Cochrane Handbook* [20].

### Data items

The extracted data included characteristics of the study and participants, details regarding interventions, outcome measures, duration until follow-up assessments, compliance, subject numbers, withdrawals, and intention to treat (ITT) analyses. For the purpose of this review, ITT analyses were defined as a complete assessment of outcomes of all randomized trial participants, regardless of withdrawal from the trial or adherence to the treatment regime [46]. This approach was taken because, without ITT analyses, there is potential for overoptimistic results of intervention efficacy [47].

### Risk of bias in individual studies

The internal validity of the included trials was independently appraised by the two reviewers using the Cochrane Collaboration’s tool for assessing risk of bias [20]. This tool was used as each of its domains is supported by empirical research, as is the use of the component rather than the scale approach [20,48]. This flexible, adaptive tool also fits well where the intervention cannot be blinded, as the potential influence of findings is considered in the appraisal. The reviewers used standardized forms to document each component of the risk-of-bias appraisal, and inter-observer agreement between reviewers was evaluated using Cohen’s weighted *κ* [44], again using the Landis and Koch criteria for interpretation [45]. If reviewers disagreed, agreement was sought by consensus after consulting the Cochrane tool guidance [20], with a third reviewer mediating in the event of on-going disagreement. Risk-of-bias scores were summarized in line with Rushton et al. [49]. The study selection and risk of bias assessment processes were initially piloted by the two reviewers.

### Summary measures

If feasible, a common measure of injury risk reduction would have been used to facilitate data synthesis across trials with similar interventions.

### Synthesis of results

Comparability of interventions, outcomes and timing of assessments, and risk of bias were considered to determine potential for appropriate quantitative synthesis of the trials. If the trials were sufficiently comparable and had a low risk of bias, meta-analyses would be performed and if not, a qualitative data synthesis produced.

### Risk of bias across studies

A summary of risk of bias across trials was tabulated, and the overall risk of bias was agreed by consensus. Differences in trial design, intervention, and outcome, and the inclusion of fewer than ten trials rendered it not helpful to produce a funnel plot to visualize potential publication bias [20,34].

### Additional analyses

If at least two trials were available with comparable study design, interventions, and outcomes, subgroup analyses would have been used to examine the implications of heterogeneity.

## Results

### Study selection

**Figure 1:**
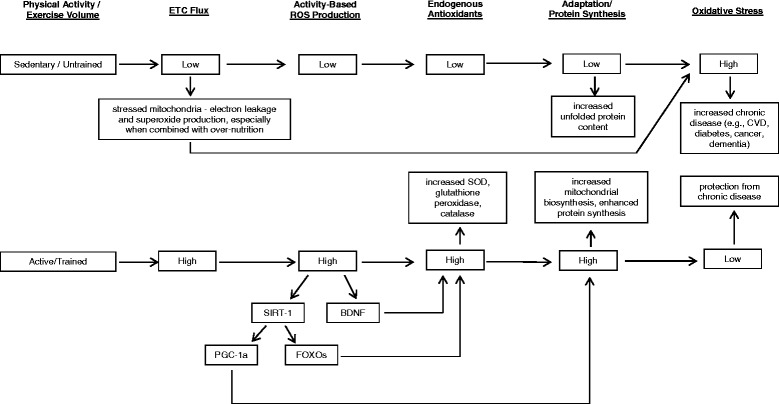
**Study selection flow diagram [** 22 **].**

Eight trials from five countries were included [12,50-56]. Database and hand searches provided a total of 1,942 citations. After removal of duplicates, 763 remained. Following screening of titles and abstracts, 744 studies were excluded as they did not meet the eligibility criteria. Of the remaining 19 studies, attempts to obtain the full texts of two unpublished studies were unsuccessful. The full texts of the remaining 17 studies were reviewed. Of these, nine did not meet the inclusion criteria. The study selection flow diagram shows the number of studies at each stage of selection (Figure 1). Using weighted *κ*, substantial inter-reviewer agreement was achieved on trial inclusion (Cohen *κ* 0.638, 95% confidence interval [CI] −0.003 to 1.280) [44,45]. Less than perfect agreement arose from the evaluation of one study due to the definition of injury. The study was included after discussion [51].

### Trial characteristics

#### Methods

Five trials investigated an exercise intervention group compared to a control group [50-54]. Two trials assessed exercise or orthotic intervention groups compared with a control group [55,56]. One trial divided participants into high- or low-risk subgroups for specific injuries and assigned the high-risk players to either an injury-specific exercise intervention group or a control group [12]. All control groups continued with their usual warm-up and training. Table 2 shows the main characteristics of the included trials.

**Table 2:** **Summary of trial characteristics**

**References country**	**Primary outcome measure/means of reporting**	**Participants**	**Intervention**	**Follow-up period**	**Compliance**	**Effect of intervention (95% CI)**	**Conclusion**	**Comments**
Askling et al. [52] Sweden	Time loss Injuries to the hamstring% Injury occurrence	IG: 15 CG: 15 Mean age IG: 24 CG: 26	10 weeks preseason intervention encompassing 16 sessions of hamstring training using concentric and eccentric actions, after a standardized warm-up	1 season of 10 months	NR	IG 20% CG 67% Significantly fewer injuries in the IG (*p* < 0.05)	Eccentric hamstring training may result in a lower occurrence of hamstring strains	High injury rate in the CG (67%) No loss to FU
Engebretsen et al. [12] Norway	Time loss Injuries to the ankle, knee, groin, or hamstring in high-risk players Mean injury Incidence per 1,000 h/RR)	High-risk players IG: 193 CG: 195 Mean age NR	Progressive exercise program for the ankle, knee, groin, and/or hamstring 3 × a week for 10 weeks then × 1 a week for the rest of the season	1 season of 7 months	19–30% during the preseason intervention	IG = 4.9 CG = 5.3 RR 0.93 (0.71–1.21) This was NS (*p* = 0.50)	The risk of injury in players deemed at higher risk was not changed with a targeted training program	19 of the 31 teams already performed preventive exercises in regular training ITT analysis conducted 3% players lost to FU
Fredberg et al. [51] Denmark	All injuries to the Achilles and/or patellar tendon% Injury risk/RD	IG: 98 CG: 146 Mean age 25	Eccentric exercise and stretching program of Achilles and patellar tendons All exercises performed for 10 min 3 times a week	12 months	2.25/week	Patella = RD 0% (*p* = 1.0) Achilles = RD 2% (*p* = 0.86) These were NS	Eccentric exercise had no positive effects on the risk of Achilles or patella tendon injury	CG ‘allowed to continue with the different types of flexibility training that they all used’ No ITT analysis 17% team withdrawn
Hölmich et al. [53] Denmark	All injuries to the groin Time to first groin injury/HR	IG: 524 CG: 453 Mean age IG: 24 CG: 25	6 exercises including hip adduction and abdominal strengthening, coordination and stretching, 2–4 times a week	1 season of 10 months	NR	HR 0.69 (0.40–1.19) This was NS (*p* = 0.18)	Intervention resulted in no significant reduction in groin injury risk	No ITT analysis Injured players censored 56% teams and 7% of remaining players withdrawn
Mohammadi [55] Unclear	All ankle inversion sprain re-injuries RR of injury per 1,000 h	Each IG: 20 CG: 20 Mean age 25	Progressive ankle disc training for 30 min daily (PT) Isometric and dynamic specific evertor strength training (ST).	1 season after injury	NR	PT − RR 0.13 (0.003–0.93) significantly fewer injuries in the IG (*p* = 0.02) ST − RR 0.5 (0.11–1.87) This was NS (*p* = 0.27)	Progressive ankle disc training may reduce the risk of recurrent ankle inversion injuries	Information on much of the study design is lacking No ITT analyses No loss to FU
Petersen et al. [50] Denmark	All hamstring injuries Injury rates per 100 player seasons/rate ratio	IG: 461 CG: 481 Mean age IG: 23 CG: 24	10 weeks of progressive eccentric hamstring training followed by a weekly program	1 season (2 half seasons over 12 months)	91% of the initial 27 sessions	IG 3.8 CG 13.1 Rate ratio 0.292 (0.136–0.631) Significantly fewer injuries in the IG (*p* < 0.001)	Additional eccentric hamstring training significantly reduced the risk of hamstring strain	No ITT analysis 7% teams and 8% of remaining players withdrawn
Tropp et al. [56] Sweden	Time loss ankle sprains in those with a history of sprain% Injury risk	CG: 75 IG: 65 Mean age NR	10 min of ankle disc training 5 times a week for 10 weeks. Then 5 min, 3 times a week	6 months	NR	IG 5% CG 25% Significantly fewer re-injuries in the IG (*p* < 0.01)	In players with a history of ankle sprain, ankle disc training is indicated to reduce the risk of re-injury	Information on key aspects of study design lacking No loss to FU
van Beijsterveldt et al. [54] Holland	All injury incidences Injury incidence per 1,000 sports h	IG: 223 CG: 233 Mean age IG: 24 CG: 25	10 exercises, used at each training session, 2/3 times a week. Included core stability, muscle strengthening, proprioceptive, stabilization and plyometric exercises	1 season of 9 months	71% player compliance	IG: 9.6 (8.4–11.0) CG: 9.7 (8.5–11.1) This was NS (*p* > 0.05)	No significant differences found in the overall injury incidence or injury severity between the IG and CG	No ITT analysis 6% players lost to FU

*CG* control group, *CI* confidence interval, *FU* follow-up, *HR* hazard risk, *IG* intervention group, *ITT* intention-to-treat analysis, *NR* not reported, *NS* not significant, *RD* risk difference, *RR* relative risk, *ST* strength training, *PT* proprioceptive training.

#### Participants

The eight trials included a total of 3,355 participants meeting the eligibility criteria. Five trials [50-54] and 2,647 participants had no reported history of injury or increased risk of injury; 200 participants with a history of injury were recruited in two trials [55,56]. A further trial included assessment of 388 participants reported to be at high risk of injury [12].

#### Interventions

A range of exercise interventions were used, including specific targeted muscle strengthening and/or stretching [50-52,55], proprioceptive exercise [55,56], and multi-faceted exercise intervention regimes [12,53,54]. Most trials used supervised exercise intervention as part of or in addition to regular training [12,50,51,53,54]. One trial used four supervised and then largely independent exercise sessions [52].

#### Outcomes

A range of injury types were assessed. Several trials investigated the incidence of specific injuries, including hamstring [50,52], ankle [55,56], Achilles and/or patellar tendon [51] or groin injuries [53]. Engebretsen et al. [12] assessed the incidence of injuries to the ankle, knee, groin, or hamstring, and van Beijsterveldt et al. [54] investigated all injuries.

Three trials reported a primary outcome on which a power calculation was based [50,53,54]. For each of these, the primary outcome was injury, defined as a physical complaint obtained during training or match play irrespective of the need for medical attention or time loss from football activities. This was also the main outcome of a further two trials [51,55]. The three remaining trials had a main outcome of time loss injuries, defined as an injury that results in a player missing training or match play [12,52,56].

Table 2 demonstrates that measures used for reporting injury incidence varied. The use of time until first injury [53] and total number of injured players rather than number of injuries in two trials [51,53] resulted in an inability to create a common measure of risk reduction for all of the trials. Several trials used further outcomes, including changes to athletic ability [52] and ultrasound assessment of structural changes to a tendon [51]. Other injury-related outcomes, such as severity of injury, were used by a number of trials [12,50,51,54].

### Risk of bias within studies

Using weighted *κ*, moderate inter-reviewer agreement was achieved on evaluating trial risk of bias (Cohen *κ* 0.602, 95% CI 0.402–0.788), with 100% agreement following discussion [44,45]. All eight trials were evaluated as high risk of bias (Table 3), which impacts on the interpretation of results [20].

**Table 3:** **Summary of risk of bias scores** [20]**, reported in line with Rushton et al.** [49]

**References**	**Components of risk of bias** ^**a**^						**Overall**	**Comments, high-risk components**
	**1**	**2**	**3**	**4**	**5**	**6**		
Askling et al. [52]	U	U	H	U	U	U	High 1 Unclear 5 Low 0	High-risk components: 1 No participant, intervention provider, or outcome assessor blinding
Engebretsen et al. [12]	U	U	H	L	U	U	High 1 Unclear 4 Low 1	High-risk components: 1 No participant, intervention provider, or outcome assessor blinding
Fredberg et al. [51]	U	U	H	U	U	U	High 1 Unclear 5 Low 0	High-risk components: 1 No participant, intervention provider, or outcome assessor blinding
Hölmich et al. [53]	L	U	H	U	U	L	High 1 Unclear 3 Low 2	High-risk components: 1 No participant, intervention provider, or outcome assessor blinding
Mohammadi [55]	U	U	U	L	U	H	High 1 Unclear 4 Low 1	High-risk components: 1 Inadequate reporting of many aspects of study design
Petersen et al. [50]	L	U	H	L	L	L	High 1 Unclear 1 Low 4	High-risk components: 1 No participant, intervention provider, or outcome assessor blinding
Tropp et al. [56]	U	U	U	L	U	H	High 1 Unclear 4 Low 1	High-risk components: 1 Inadequate reporting of many aspects of study design
van Beijsterveldt et al. [54]	L	U	H	L	U	U	High 1 Unclear 3 Low 2	High-risk components: 1 Insufficient participant, intervention provider, and outcome assessor blinding

^a^Components of risk of bias: 1, random sequence generation; 2, allocation concealment; 3, blinding of participants, personnel, and outcome assessors; 4, incomplete outcome data; 5, selective reporting; 6, other bias. Levels of risk of bias: H, high risk of bias; U, unclear risk of bias; L, low risk of bias.

### Results of individual studies

Two of the four trials reporting a statistically significant reduction in injury related to hamstrings. Each showed a significantly lower incidence of hamstring strain with eccentric exercise intervention. Petersen et al. [50] reported 3.8 acute hamstring injuries per 100 player seasons in the intervention group and 13.1 in the control group (rate ratio 0.292; 95% CI 0.136–0.631; *p* < 0.001); Askling et al. [52] reported 20% of players experienced hamstring injuries in the intervention group and 67% in the control group (*p* < 0.05).

Two of the four trials reporting a statistically significant reduction in injury related to ankle injuries in those with history of injury, with proprioception training [55,56]. Mohammadi [55] reported a reduced risk of re-injury in a proprioception training intervention group (relative risk of injury 0.13; 95% CI 0.003–0.93; *p* = 0.02) and no reduced risk in a strength training group (0.5; 95% CI 0.11–1.87; *p* = 0.27). Tropp et al. [56] showed that significantly fewer of the proprioception intervention group experienced re-injuries (5%) compared with the control group (25%) (*p* < 0.01).

Four trials showed no statistically significant difference in injury incidence between control and exercise intervention groups [12,51,53,54].

### Synthesis of results

All trials were of high risk of bias and were not sufficiently homogenous for meta-analysis. When grouping trials according to injury type, two trials evaluated hamstring injuries, two trials evaluated ankle sprain re-injuries, and four trials were not comparable. No further analyses were conducted owing to differences in study design, intervention, and outcomes across the trials. The two hamstring trials used different interventions, duration of intervention period, and outcomes (injury definition), and also had differences in study design, notably with use of clustering [50,52]. The ankle trials were not comparable on intervention, outcomes (injury definition), and timing of outcomes [55,56]. A qualitative analysis was conducted.

### Risk of bias across studies

Table 3 shows consistent components of risk of bias across the trials, including performance and detection bias due to inadequate blinding in all of the trials, and selection bias due to a lack of transparency regarding sequence generation in most trials [12,51,52,55,56] and of allocation concealment in all trials. There was a lack of blinding of outcome measurement, with evidence of injury registration being performed by personnel providing the intervention in all of the included trials in which the injury registration process is reported [12,50-54]. There were also issues regarding blinding of research staff, with staff visiting intervention groups during the study period in two of the trials [52,55]. Only one trial had a published protocol demonstrating pre-defined study goals [50], introducing a risk of selective reporting of outcomes in the other trials. Two trials were poorly reported, limiting accurate assessment of their internal validity [55,56]. A number of trials did not present complete outcome data, using available case analyses rather than including all randomized participants. Three of the trials had full data on all randomized subjects [52,55,56] and one of the remaining trials conducted an ITT analysis [12]. A high rate of withdrawals introduced a risk of attrition bias to the trial by Hölmich et al. [53], and the reliability of several of the trials was impaired by low cohort sizes or evidence of insufficient power [50,52,55,56]. Compliance was poorly reported and, of the four trials in which compliance with at least part of the intervention is documented [12,50,51,54], lower compliance than pre-defined targets was reported in two [12,51].

## Discussion

### Summary of evidence

This systematic review evaluated the efficacy of exercise for injury prevention in adult male football. The eight included trials, with 3,355 participants, used a range of exercise interventions and outcome measures. Disappointingly, despite a number of trials being recent, all trials were assessed as having a high risk of bias. Four trials demonstrated statistically significant reductions in the incidence of injury: two relating to hamstring strains and two relating to recurrent ankle sprains. These findings are of interest, given that hamstring and ankle injury are two areas most prone to injury in footballers [5,57].

Hamstring strains are one of the most common injuries at professional football clubs, accounting for 12–16% of all injuries [5,39,58,59]. It is believed that they often occur during rapid eccentric loading when they are stretched beyond the optimal torque for generating tension, notably during sprinting [52,58,60]. It has been suggested that eccentric exercise increases this angle of optimal torque and may reduce the risk of injury [61-65]. There was some evidence demonstrating a reduction of the incidence of hamstring injury over the course of a season as an outcome of pre-season eccentric hamstring training, with two trials showing statistically significant reductions [50,52]. Although these findings are interesting, both trials were assessed as having a high risk of bias due to inadequate blinding, notably lacking blinding of staff registering injuries. The positive findings of these trials are consistent with those evaluating male athletes from different sports [66,67]; however, these trials are also of poor quality. These findings from trials with a high risk of bias demonstrate the need for an adequately powered trial with a low risk of bias to evaluate the efficacy of eccentric hamstring exercises on hamstring injury incidence. Future research should also consider whether participants have a history of injury, as both of the included trials demonstrated a reduction of hamstring injury recurrence with eccentric training [50,52]. Notably, Petersen et al. [50] demonstrated a significant reduction of approximately 85%. This is particularly relevant given that previous injury is the greatest risk factor for hamstring strain [68] and that re-injuries cause longer absences from sport [5].

History of injury is also a key risk factor for other football injuries [69,70], including ankle injuries, which account for 11% of injuries to footballers and have a high recurrence rate of up to 34% [38,71]. The high risk of recurrence has been associated with impaired sensorimotor function after injury in the form of proprioceptive deficits, such as muscle reaction timing [72]. Balancing exercises are purported to improve these deficits [73]. There was some evidence of reduced incidence of ankle sprain re-injury as an outcome of balancing exercises using an unstable surface, with two trials showing statistically significant reductions [55,56]. Although these findings are interesting, the trials were assessed as having a high risk of bias due to poor reporting. There is not sufficient evidence to reach a clear conclusion regarding the efficacy of proprioceptive training on ankle injury recurrence in adult male football. An adequately powered trial with a low risk of bias is required, particularly considering a recent meta-analysis including male and female, athletic and non-athletic populations that found no statistically significant difference in risk of ankle re-injury with proprioceptive training [74].

Four trials, each evaluating different injuries, were unable to provide evidence of efficacy of exercise intervention for injury prevention, with each reporting no statistically significant benefit. One of these trials identified footballers at increased risk of injury, but the targeted exercise intervention did not reduce the incidence of common football injuries [12]. A major limitation of this trial was low compliance with the intervention (19–30%), which increased the risk of type II error. Groin injuries are common in football and can lead to prolonged periods away from play, hence a program to reduce the risk of groin injury could be of great benefit to football clubs [40]. However, the trial by Hölmich et al. [53] that evaluated a groin injury prevention program also showed no statistically significant effect on injury incidence. One further trial did not provide support for the prophylactic use of eccentric exercise for preventing Achilles or patellar tendon injuries [51], despite some evidence supporting the use of eccentric exercise for the treatment of tendon injuries [75]. A further finding was that a general injury prevention program evaluated in the trial by van Beijsterveldt et al. [54], which included a range of exercises in the form of a warm-up, did not show statistically significant effects on injury incidence over a season. Their finding of no effect on overall injury incidence was similar to the findings of trials using the same program, ‘The 11’ or the modified version ‘The 11+’ in adolescent female football players [76,77].

The key finding of this review is that, based on the current level of evidence, it is not possible to evaluate whether exercise is efficacious in the prevention of injuries in adult male football. A lack of high-quality RCTs of injury prevention in the adult male football population is contradictory to the number of publications on injury epidemiology and risk factors in this population. The findings are similar to those of van Beijsterveldt et al. [24], who reviewed general exercise programs for injury prevention in footballers, finding conflicting and weak evidence to support their use. However, the findings are in contrast to the systematic review by Kirkendall and Dvorak [21], which uses findings, including those from studies with male participants, to support the use of a range of exercises in an injury-prevention program. The latter review demonstrates a number of methodological flaws; hence, the findings must be extrapolated with caution. Findings of this review also contrast with those in adolescent female footballers, where positive effects of neuromuscular exercise programs on injury incidence have been found [17]. This could be due to a number of reasons, including a difference in the intervention type or compliance, or a disparity in the quantity and quality of research. It could also be due to age- or sex-related differences in study participants [28,29]. It is possible that neuromuscular programs are more effective in reducing the risk of injury in female footballers because they address neuromuscular imbalances and movement patterns that have been associated with an increased risk of severe injury, such as ACL rupture, in females [28,78]. Corresponding with this, it has been suggested that programs designed to target gender-related differences in injury risk and incidence may be warranted [28].

Two definitions of injury were used by the trials, either ‘any injury’ or ‘time loss injury’, both of which correspond with those recommended by the consensus statement published in 2006 by Fuller et al. [79]. Consensus should be found regarding the use of specific outcome measures, such as injury definition, which would allow the use of data pooling in the future. Wider reporting of injury severity and duration of time loss from football would also benefit future research in this field, allowing the impact of injuries to be assessed. Also, incidence of adverse effects should be more widely reported, as demonstrated by the findings of Fredberg et al. [51] where, contrary to expectation, eccentric exercise increased patella tendon injury incidence in footballers with degenerate tendons.

### Strengths and limitations

Strengths of this review are its rigorous methodology based on a pre-defined protocol. Its main limitation was the finding of poor reporting of included trials and high risk of bias components. These findings are particularly disappointing considering a number of trials were published after the Consolidated Standards of Reporting Trials (CONSORT) statement, which supports explicit reporting of trials [80,81]. High risk of bias components included randomization and concealment of allocation, processes that, if inadequately performed, may lead to an overestimation of treatment effect [82,83]. The nature of the interventions may preclude blinding of the participants; however, this also introduced a risk of bias, particularly with the use of subjective measures such as self-reporting of injury [84] as used in the trials by Askling et al. [52] and Fredberg et al. [51]. There was also little evidence of predefined outcomes, partly due to a lack of published protocols. Issues relating to compliance are also notable, as direct correlations between compliance and the efficacy of injury prevention programs have been found [85].

Using GRADE [86] (the Grading of Recommendations Assessment, Development and Evaluation system), based on the eight trials included in the qualitative synthesis, the quality of evidence evaluating the efficacy of exercise intervention on injury prevention in adult male football was ‘very low’. This level of evidence is defined as “very little confidence in the effect estimate: the true effect is likely to be substantially different from the estimate of effect” [86]. The quality of evidence was downgraded due to high risk of bias, inconsistent results, indirectness of evidence, and imprecision [86].

## Conclusions

This rigorous systematic review has identified very low-quality evidence on the efficacy of exercise on prevention of injuries in adult male football. There is some support for the use of eccentric hamstring exercise for hamstring injury prevention and proprioceptive training to reduce the incidence of recurrent ankle sprains; however, sufficient evidence is lacking to reach a clear conclusion regarding their efficacy. There is a need for low risk of bias, comprehensively reported and properly powered trials evaluating the efficacy of generic and specific exercise interventions for injury prevention. Notable areas of future research may relate to the prevention of ankle and hamstring injuries. Future research should also be considered regarding the efficacy of exercise in preventing groin injuries and knee sprains, for these are also major injury burdens in male adult football and lack high-quality trials evaluating efficacy of preventive interventions [4,5,12,69].
